# Clinical care & blood pressure control among hypertensive people living with human immune deficiency virus: Prospective cohort study

**DOI:** 10.1016/j.amsu.2020.04.017

**Published:** 2020-05-05

**Authors:** Tsegaye Melaku, Legese Chelkeba, Zeleke Mekonnen

**Affiliations:** a School of Pharmacy, Institute of Health, Jimma University, P.O.Box: 378, Jimma, Ethiopia; b School of Medical Laboratory, Institute of Health, Jimma University, P.O.Box: 378, Jimma, Ethiopia

**Keywords:** Human immune deficiency virus, Blood pressure control, Treatment outcome, Hypertension, Self-care

## Abstract

**Background:**

Hypertension has emerged as a new threat to the health and well-being of people living with human immune deficiency virus (PLHIV). However, no data exist on care delivery and blood pressure control over time in Ethiopia. We assessed clinical care & level of blood pressure control among hypertensive people living with Human Immune Deficiency Virus (HIV).

**Methods:**

We conducted a prospective cohort study among adult hypertensive PLHIV and HIV-negative patientsat chronic care clinics of Jimma University Medical Center in Ethiopia. We explored self-management practices and blood pressure control of study participants. Multivariable Cox-regression was used to identify the predictors of the outcome.

**Results:**

A total of 303 eligible participants with mean age of 43.30 ± 12.55years were followed and males comprised of 52.1%. After 12 months of follow-up, 60.2% of HIV-positive and 53% of HIV-negative patients showed uncontrolled blood pressure. The overall perception of self-management behaviors was 2.10 ± 0.77 (p = 0.122), which was at moderate level. An increased waist circumference [AHR: 2.16; 95% CI: (1.58–5.18);p = 0.021],chronic disease co-morbidity[AHR:3.94;95%CI:(2.24–8.74);p = 0.046],alcohol use history[AHR:1.26; 95%CI:(1.08–2.23);p = 0.031], HIV infection[AHR:3.06;95%CI:(1.93–11.34);p=0.042], infrequent use of fruits & vegetables [AHR:3.77;95%CI: (1.34–10.57);p=0.012], infrequent engagement on physical exercise[AHR:3.48;95%CI:(1.48–8.17);p = 0.004],frequent use of high fats food [AHR:2.56;95%CI: (1.25–5.25);p = 0.011] were an independent predictors of uncontrolled blood pressure.

**Conclusion:**

The rate of uncontrolled blood pressure is significantly higher in the HIV- infected population. There was a gap in the clinical care of hypertension in terms of hypertension self-management among hypertensive HIV-positive patients. Our study highlights the need for better integration of hypertension care to HIV clinical setting.

## Highlights

• Integration of hypertension care within the HIV clinical setting will maximize care efficiency & synergy.
• The rate of uncontrolled blood pressure is significantly high among PLHIV.
• There was a gap in self-management and clinical care of hypertensive PLHIV.
• HIV infection posed a statistically significant hazard on blood pressure control.

## 1 Introduction

Cardiovascular disease is becoming one of the leading causes of morbidity and mortality in patients living with human immune deficiency virus (HIV). Hypertension is the most prevalent modifiable risk factor for cardiovascular disease. Since its prevalence is higher than in the general population, the diagnosis, management and blood pressure control is now an important part of care for these patients. For example, a study in an urban HIV outpatient clinic in New York City found that 43% of patients had hypertension with 75% treated, but only 57% of these were controlled [1]. A longitudinal study from the USA showed that HIV-infected persons on clinical care from 1994 to 2011 had a 20.4% incidence compared to 20.7% of HIV-negative patients [2].

**Diagram 1:**
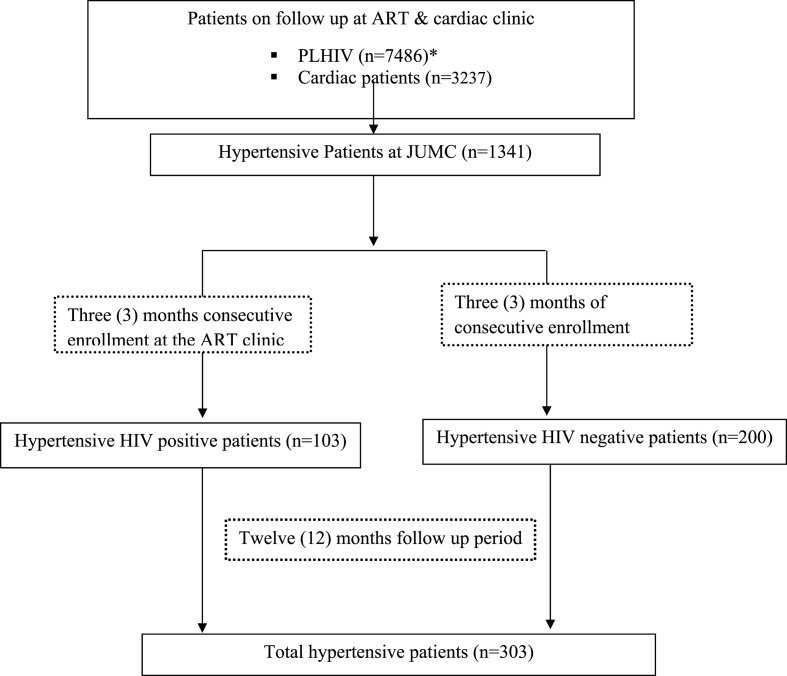
Study participants' enrollment flow chart.

Currently, there are 37 million people are living with HIV across the globe [3]. From early 2000, there is a significant increase in the number of people living with HIV (PLHIV) who get access to antiretroviral therapy (ART) [3,4]. According to the WHO report, within 10 years (from 2005 to 2016) HIV-related mortality rate has halved from 2 million deaths to 1 million due to widespread ART use [4]. However, paradoxically during the same period, cardiovascular disease mortality rates more than doubled in PLHIV [5].

Hypertension is the leading risk factor for cardiovascular mortality across the world. It is also a growing problem in HIV-infected adults [6]. As HIV-infected adults on ART have a higher prevalence of hypertension when compared with HIV-uninfected individuals, the morbidity and mortality are expected to be higher [7]. A meta-analysis done by Wang K et al. [8] showed that 35% of all HIV-infected adults on ART have hypertension, compared with an estimated 30% of HIV-uninfected adults. Among ART-experienced individuals over 50 years of age, >50% of them have hypertension [8].

Besides, hypertensive HIV-infected have a higher risk of cardiovascular events and all-cause mortality than HIV-uninfected adults with hypertension or normotensive HIV-infected adults [9,10]. A prospective cohort study of HIV-infected and uninfected veterans in America found that HIV-infected adults with hypertension had a 2-fold higher risk of incident acute myocardial infarction as compared with HIV-uninfected adults with hypertension [11].

Risk factors for hypertension in the general population are similar to those in patients infected with HIV. These include older age, sensitivity to salt, genetic predisposition, and lifestyle factors such as overweight and obesity, smoking, excessive alcohol, and being sedentary [12]. The etiologies in patients living with HIV are more complex. There is felt to be a greater frequency of traditional risk factors [1] as well as factors unique to HIV-infection. Some research suggests that body composition, inflammation, and immune activation may influence blood pressure [13,14].

Given the overall highburden of both hypertension and HIV infection in Africa, it is crucial to determine the hypertension self-management practices and blood pressure control in the HIVinfectedpopulation. This will help optimize health-care delivery to this vulnerable group. This is particularly pertinent today with the widespread implementation of antiretroviral therapy (ART), which has significantly prolonged the life spans of HIV-infected individuals [15,16]to the extent that these are now comparable with the general population. Successful strategies for hypertensive management will ultimately depend upon the patient's self-management, or the ability and willingness of the patient to change and maintain certain behaviors. In this study, our conceptualization of hypertensive self-management is based on the work of Lin et al. [17]and the related literature about hypertension treatment care. The outcome of the self-management process is individual behaviors intended to maintain or improve health and prevent exacerbation [18].

Chronic non-communicable diseases (NCDs) co-morbidity in HIV infection, the occurrence of an NCD in an individual with HIV, currently requires more attention along with the rapid emergence of NCDs as a major disease of public health importance in high HIV prevalent areas. Low-and-middle income countries (LMICs), like Ethiopia, already have a high magnitude of HIV and are expected to share a high burden of hypertension co-morbidity due to the associated increase in the incidence of non-communicable diseases. Little is known about the effect of HIV infection and its treatment of hypertension care and blood pressure control.

To date, there is no single study done in Ethiopia and as well a few and less robust data from some African countries to investigate the nature and effect of HIV on blood pressure control. Surprisingly, care for both HIV/AIDS and hypertension has been provided independently, despite several hypertensives HIV infected patients on follow up. To our knowledge, the present study is the first in the literature to evaluate the response to antihypertensive therapy in patients with HIV infection compared to those without HIV infection in sub-Saharan regions. This study will further inform the need to get comprehensive clinical services i.e. launching comprehensive chronic care clinics (CCCC) that have continuity, through the integration of the two cares to maximize efficiency and synergyforhypertensive HIV patients.

## 2 Methods

### 2.1 Study design and setting

A hospital-based prospective cohort study was conducted at chronic care (ART and ambulatory) clinics of Jimma University Medical Center (JUMC), Ethiopia. JUMC is the only teaching and referral hospital in the South-Western part of Ethiopia with a bed capacity of 660. Geographically, it is located 352 km South-West of Addis Ababa, the capital. It provides services for approximately 9000 inpatient and 80,000 outpatient clients per year with a catchment population of about 15 million people. The hospital has a separate ART clinic with about 7486 clients on follow up. The services include HIV care and treatment, TB treatment, and prevention of mother to child transmission services. There are also separate ambulatory clinics for the management of cardiac disease, neurologic disease, diabetes mellitus, and psychiatric disorders in the setting. The work has been reported in line with the strengthening the reporting of cohort studies in surgery (STROCSS) criteria [19].

### 2.2 Study participants

This current study was a comparative prospective cohort study assessing arterial hypertension clinical care and the level of blood pressure (BP) control between two groups of the population. The first group was adult hypertensive people living with HIV (PLHIV) on follow-up and receiving HAART at the ART clinic of JUMC. The second group was adult hypertensive HIV negative individuals on follow up for hypertension clinical care at the ambulatory clinic of JUMC.

### 2.3 Participants inclusion and enrollments

In the study area, the number of PLHIV and diagnosed with hypertension was unknown and had a separate follow-up clinic and day of consultation. All adult PLHIV, who were coming for HAART refill and consultation during the data collection period were interviewed and their medical record (of HIV and hypertension) were reviewed. The patient with reported hypertension diagnosis was cross-checked for the information at their respective follow-up clinic. Patients on ART care and treatment in the settings had followed up a period of one month to three [3] months in the setting. The enrollment period of patients to the study cohort took 3 months (December 2018 to February 2019) to get all potential study participants. And finally, 103 hypertensive adults PLHIVwere included in the study and assessed for their blood pressure control status and clinical care. Similarly, adult HIV negative hypertensive patients on follow up were enrolled inthe study. To increase the power of the study, we enrolled 200 hypertensive HIV negative participants(~ratio1: 2). Finally, 303 hypertensive participants were included in the final analysis. All the participants were followed for 12 months starting from their enrollment date (Diagram 1).

### 2.4 Data collection tool and procedure

A structured data collection questionnaire developed by the World Health Organization(WHO) on a stepwise approach to chronic disease risk factor surveillance [20,21] was used with modifications according to the study objective. Other quantitative data were also collected through patient self-report using a structured questionnaire on patient self-management practice and behavior in hypertension management [22]. Two trained data collectors (both ART trained nurses) interviewed the study participants and reviewed patient charts and medical records for the respective information at the ART clinic. Another two trained research assistants (nurse in the profession) followed the HIV negative hypertensive patients for 12 months at the cardiac clinic of JUMC. All the information such as baseline socio-demographic data, clinical and laboratory information and behavioral characteristics of each patient were recorded. Patients were also clinically examined and measurements were taken for weight, height, waist circumference and BP. A one year monthly records of BP records at follow up clinic were recorded for each patient. An anthropometric measurement [waist circumference (WC)] was measured with a flexible inelastic tape placed on the midpoint between the lower rib margin and the iliac crest in a perpendicular plane to the long axis of the body. Height was determined without shoes using a portable stadiometer. Weight was measured using a Tanita scale; patients were fully dressed, without heavy clothing or shoes.

### 2.5 Outcome measures and validation methods

#### 2.5.1 Blood pressure

The BP of all hypertensive [both HIV positive (HIV (+)) and HIV negative (HIV (−))]patients coming for follow up to clinics every month was measured according to the usual methods of the institution. No recommendations or training were provided for BP measurement. This is not to interferewith the usual trends for service provision by recommending BP measurement and patient health education. The data collectors collect all the parameters from ART and/or cardiac clinics.When BP of HIV positive patients not recorded at the ART clinic, it was traced back from cardiac clinic medical record and patient her/himself. A serial of 12 months of BP records was documented and its status was categorized into controlled BP or uncontrolled BP based on the seventh (7th) joint national commission guideline [23].

#### 2.5.2 Self-management behavior

The hypertension self-management behavior questionnaire (HSMBQ) consisted of 40 items addressing the five components of self-management. These include self-integration (13 items), self-regulation (9 items), interaction with the health professional and significant others (9 items), self-monitoring (4 items) and adherence to the recommended regimen (5 items). The subjects were asked to rate each item to indicate the frequency in which they performed the self-management practices. Items were scored on a 4- point scale ranging from 1(never) to 4(always). The self-management scores were divided into three levels: low, moderate and high. The scores of 1.00–2.00 means a low level of self-management; scores of 2.01–3.00 mean a moderate level of self-management and scores from 3.01 to 4.00 means a high level of self-management. The internal consistency and reliability of the self-management instrument were assessed by using Cronbach's alpha 0.80.

Self-integration refers to a patient's ability to integrate hypertensive care into their daily lives through activities such as proper diet, exercise, and weight control. Self-regulation reflects the patient's self-regulation about their behaviors through self-monitoring of body signs and symptoms. This involves life situations and causes related to changes in blood pressure and taking an action based on these observations. Interaction with health professionals and significant others is based on the concept that good blood pressure control care involves collaboration with health care providers and significant others. Self-monitoring is concerned with the monitoring of blood pressure for detecting blood pressure levels to adjust self-care activities and adherence to recommended regimens. In turn, this is related to the patient's adherence to prescribed antihypertensive medication and visits to clinics.

### 2.6 Data processing and analysis

Data were entered into the computer using EpiData version 3.1 and exported to the Statistical Package for Social Science (SPSS) version 22.0 (IBM, Armonk, NY, USA)for analysis. Descriptive statistics [frequency, percentage, and means ± standard deviations (SD)] were used for reporting of patient characteristics, clinical data, behavioral and related factors. Categorical and continuous data were expressed as percentages and mean ± standard deviation, respectively. Chi-square (χ2) test was used to test the significance of associations between categorical variables. Cox-regression analyses were used to assess the crude and adjusted effect of seemingly significant predictors of blood pressurecontrol. Variables that had p-value≤ 0.25 on univariate analysis were eligible for multivariate Cox-regression. Two-sided P values < 0.05 were accepted as significant.

### 2.7 Ethical approvaland consent to participate

Ethical clearance & approval was obtained from the institution review board (IRB) of Jimma University. The data that was collected from the JUMC chronic care clinic was preceded by a formal request letter from Jimma University. Written informed consent was taken from each study participant after a clear orientation of the study objective. The raw data were not made available to anyone and not used as the determinant of the participant. Strict confidentiality was assured through anonymous recording and coding of questionnaires and placed in a safe place. The patient got full right not to participate and as well as leave the study at any time during the study time. The study was registered researchregistry.com with a unique reference number of ‘’researchregistry5258’’

### 2.8 Definition and explanations of terms

⁃ *Hypertension:* sustained high blood pressure (BP) (SBP ≥ 140 or DBP ≥ 90 mmHg) with reported regular use of antihypertensive medication(s) [24].
⁃ *Uncontrolled BP:* was defined as SBP of ≥140 mmHg and/or DBP of ≥90 mmHg [24].
⁃ *Controlled BP:*SBP of <140 mmHg and/or DBP of <90 mmHg [24].
⁃ *Rate of BP control:* The number of participants with controlled BP divided by the total number of participants.
⁃ *Co-morbidity:* Diseases or disorders that exist together with an index disease or co-occurrences of two or more diseases or disorders in an individual.
⁃ *Chronic non-communicable disease:* Diseases which cannot be transmitted to other through contact from the index person and not caused by disease-causing microorganisms, and patients are on follow-up for care and treatment at health institution at least for the last 30 days.
⁃ *Multi-morbidity:* Living with two or more types of chronic non-communicable diseases.
⁃ *Self-management:* ‘’an active, flexible process in which patients develop strategies for achieving desired goals by regulating their actions, collaborating with health care providers and significant others, and performing preventive and therapeutic health-related activities'' [17]. The term “self-management” also refers to the activities people undertake to create order, discipline, and control in their lives [26].
⁃ *Chat/Khat:* The khat plant (Catha edulis Forsk) is a tree of the family Celastraceae that is frequently cultivated in certain areas of East Africa [27]. They are chewed daily by a high proportion of the adult population in some parts of Ethiopia, especially around the study area for the pleasant mild stimulant effect.
⁃ *Chat chewer:* Current user of chat plant daily

## 3 Results

### 3.1 Socio-demographic characteristics of participants

From a total of 303 patients enrolled in the study, all of them had finished the 12 months of follow up period. Male gender accounted for 58.3% of the HIV (+) group and 49% of the HIV (−) group. The mean (±SD) age was 42.13 ± 11.17 years for HIV (+) and 50.76 ± 12.64 years for HIV (−)participants. The majority of the study participants were married [176(58.1)], live in a rural area [176(58.1)], unemployed [84(27.7)], and have no regular income [199(65.7)]. The mean (±SD) of the current body mass index (BMI) was 20.11 ± 3.13 and 23.96 ± 2.91 for HIV (+) andHIV (−) participants, respectively (Table 1).

**Table 1:** Baseline socio-demographic characteristics of study participants.

Variables		Total	HIV (+)	HIV (−)	P-value
Gender	Male	158(52.1)	60(58.3)	98(49)	0.124
	Female	145(47.9)	43(41.7)	102(51)	
Age (years)	Mean ± SD	43.30 ± 12.55	42.13 ± 11.17	50.76 ± 12.64	0.136
	18–35	46(15.2)	21(20.3)	25(12.5)	
	36–50	125(41.3)	43(41.8)	82(41)	
	51–65	95(31.4)	25(24.3)	70(35)	
	≥66	37(12.1)	14((13.6)	23(11.5)	
Residence	Rural	176(58.1)	56(54.4)	120(60)	0.346
	Urban	127(41.9)	47(45.6)	80(40)	
Initial weight(mean ± SD) (kg)		64.25 ± 9.13	62.19 ± 8.57	67.86 ± 10.28	0.132
Current weight(mean ± SD)(Kg)		65.22 ± 9.56	61.43 ± 7.372	68 ± 9.495	0.067
Current BMI(mean ± SD) (kg/m^2^)		21.89 ± 4.57	20.11 ± 3.13	23.96 ± 2.91	0.063
Waist Circumference(cm)	Mean ± SD	89.5 ± 11.70	88.6 ± 11.51	90.5 ± 11.73	0.031
	Normal	201(66.3)	68(66)	133(66.5)	
	Above the normal	102(33.7)	35(34)	67(33.5)	
Marital status	Single	37(12.2)	17 (16.5)	20(10)	<0.001
	Married	176(58.1)	37 (35.9)	139(69.5)	
	Divorced	51(16.8)	26(25.3)	25(12.5)	
	Widowed	39(12.9)	23(22.3)	16(8)	
Educational status	Cannot read &write	87(28.7)	23(22.3)	64(32)	<0.001
	Primary school	133(43.9)	35(33.9)	98(49)	
	Secondary school	48(15.8)	27(26.3)	21(10.5)	
	College and above	35(11.6)	18(17.5)	17(8.5)	
Monthly income (ETB)	No regular income	199(65.7)	51(49.5)	148(74)	<0.001
	Regular income	104(34.3)	52(50.5)	52(26)	
Job/Occupation	Government employee	27(8.9)	13(12.6)	14(7)	0.178
	Non-gov't employee	13(4.3)	6(5.8)	7(3.5)	
	Self-employed	179(59.1)	61(59.3)	118(59)	
	Unemployed	84(27.7)	23(22.3)	61(30.5)	
Living situation	Living with immediate family	234(77.2)	71(68.9)	163(81.5)	0.167
	Living with extended family	52(17.2)	24(23.3)	28(14)	
	Living alone	14(4.6)	5(4.8)	9(4.5)	

BMI: Body mass index; ETB: Ethiopian Birr; HIV: Human immune deficiency virus; SD: standard deviation.

### 3.2 Behavioral characteristics of participants

Concerning behavioral characteristics of study participants, about 6.8% of hypertensive PLHIV reported taking alcohol regularly and about 20.4% of them were current smokers. Among HIV (−) hypertensive the regular alcohol user was about 4.5% and current smokers were 22.5%. Only 8.7% of HIV (+) and 16.5% of HIV (−) hypertensive individuals were consuming fruits and vegetables every day (Table 2).

**Table 2:** Baseline behavioral characteristics of study participants.

Variables		Total	HIV (+)	HIV (−)	P-value
Alcohol use	Never	234(77.3)	61(59.2)	173(86.5)	<0.001
	Occasionally	53(17.5)	35(34)	18(9)	
	Regularly	16(5.2)	7(6.8)	9(4.5)	
Smoking status	Non-smoker	187(61.7)	63(61.2)	124(62)	0 .866
	Current smoker	66(21.8)	21(20.4)	45(22.5)	
	Ex-smoker	50(16.5)	19(18.4)	31(15.5)	
Chat chewer		67(22.1)	27(26.2)	40(20)	0.231
Frequency of food with high sugar use per week	Never/rarely	82(27.1)	37(35.9)	45(22.5)	0.0003
	Two times	131(43.2)	29(28.2)	102(51)	
	Three times	37(12.2)	11(10.7)	26(13)	
	Four times	43(14.2)	19(18.4)	24(12)	
	Every day	10(3.3)	7(6.8)	3(1.5)	
Frequency of fruits& vegetables use per week	Never/rarely	11(3.6)	7(6.8)	4(2)	0. 0006
	Two times	92(30.4)	43(41.7)	49(24.5)	
	Three times	93(30.7)	25(24.3)	68(34)	
	Four times	62(20.5)	16(15.5)	46(23)	
	Every day	42(13.8)	9(8.7)	33(16.5)	
Frequency of foods in high-fat use per week	Never/rarely	104(34.3)	41(39.8)	63(31.5)	0.025
	Two times	121(39.9)	29(28.2)	92(46)	
	Three times	48(15.8)	21(20.4)	27(13.5)	
	Four times	23(7.6)	9(8.7)	14(7)	
	Every day	7(2.4)	3(2.9)	4(2)	
Frequency of physical exercise per week^a^	Never/rarely	51(16.8)	17(16.5)	34(17)	0.211
	Two times	99(32.7)	35(34)	64(32)	
	Three times	66(21.8)	27(26.2)	39(19.5)	
	Four times	45(14.8)	12(11.6)	33(16.5)	
	Every day	39(12.9)	9(8.7)	30(15)	

a It is based on 30 min of aerobic exercise per day.

### 3.3 Clinical characteristics of participants

From a total of hypertensivePLHIV (n = 103), about 47.6% of them were diagnosed with HIV three [3] years back. At baseline, about 65% of them were within the WHO clinical stage III and IV. Two-third had a previous history of TB treatment. Concerning prophylactic medication use, about 11.7% and 80.6% were on isoniazid preventive therapy (IPT) and cotrimoxazole preventive therapy (CPT), respectively.About 30.1% of PLHIV were diagnosed with hypertension before HIV infection (Table 3).

**Table 3:** Baseline clinical characteristics of HIV positive study participants.

Variables		N (%)
Time since HIV diagnosis	<12 months	13(12.6)
	12–36 months	41(39.8)
	≥36 months	49(47.6)
WHO clinical stage	I + II	36(35)
	III + IV	67(65)
CDC T-stage	I + II	86(83.5)
	III + IV	17(16.5)
Functional status	Working	84(81.6)
	Ambulatory	19(18.4)
Previously treated for TB		67(65)
Type of TB	Pulmonary TB	43(64.2)
	Disseminated TB	15(22.4)
	Not known	9(13.4)
Percentage of current IPT user		12(11.7)
The reason not using IPT	Contraindication	4(4.4)
	Finished the course of treatment^b^	6(6.6)
	Adverse drug reactions	3(3.3)
	Treated for TB recently^a^	37(40.7)
	Not known	41(45)
Percentage of current CPT user		83(80.6)
The reason not using CPT	Allergic reaction	3(15)
	CD4^+^ cell count greater than the recommended CD4 cell count	9(45)
	Pill burden	5(25)
	Not known	3(15)
Time hypertension diagnosed	Before HIV infection	31(30.1)
	After HIV, but not started HAART	18(17.5)
	After HIV, but started HAART	24(23.3)
	Not sure	30(29.1)

a Within the past 3 years.

b Within the past 6 months; CDC center of disease control, TB; Tuberculosis, WHO: world health organization; IPT: Isoniazid preventive therapy; CPT: cotrimoxazole preventive therapy.

About 12.6% of hypertensive PLHIV had a family history of hypertension (84.6% of them were first degree relatives). Among HIV (−) hypertensive patients about 38% of them had a family history of hypertension (p = 0.056). More than one-third of HIV (−) hypertensive patients were hospitalized and/or visit clinics during the follow up for acute case management. About 9.2% of study participants had chronic disease comorbidity. The most common co-morbidity in the patients was a renal disease (42.7%). Concerning therapeutic life change advises related hypertension, most of the individuals received information about the medication they use for hypertension (98.02%), salt reduction (89.4%), and exercise (63.04%). However, only 41.7% of HIV (+) hypertensive patients received advice on the use of the DASH diet by health care professionals. Enalapril 191 [63] and amlodipine 132(43.6%) were the most commonly prescribed antihypertensive medication. More than two-third, 207(68.3), of the participants, were prescribed with dual antihypertensive and 120(39.6) were prescribed with thiazide diuretics (hydrochlorothiazide) (Table 4).

**Table 4:** Baseline clinical information related to the high blood pressure of study participants.

Variables		Total	HIV (+)	HIV (−)	P-value
Have a family history of hypertension		89(29.4)	13(12.6)	76(38)	0.056
Family category with hypertension	First degree relatives	56(62.9)	11(84.6)	45(59.2)	0.531
	Second-degree relatives	33(37.1)	2(15.4)	31(40.8)	
Age hypertension diagnosed (years)	<40	121(39.9)	51(49.5)	70(35)	0.344
	≥40	182(60.1)	52(50.5)	130(65)	
Hospitalization during follow up^¥^		111(36.6)	42(40.7)	69(34.5)	0.283
Hospitalization frequency	One time	80(72.1)	36(85.7)	44(63.8)	0.341
	More than times	31(27.9)	6(14.3)	25(36.2)	
Presence of co-morbidity***		89(29.4)	47(45.6)	42(21)	0.327
Other co-morbidity	Diabetes mellitus	28(31.5)	7(14.9)	21(50)	0.152
	Heart failure	9(10.1)	5(10.6)	4(9.5)	
	Dyslipidemia	5(5.6)	2(4.3)	3(7.1)	
	Epilepsy	11(12.4)	3(6.4)	8(14)	
	Renal diseases	38(42.7)	8(17)	30(71.4)	
	Other **	6(6.7)	0	6(14.3)	
Advice for high blood pressure by HCP	Drugs (medication) use	297(98)	98(95.1)	199(99.5)	0.032
	Reduce salt intake	271(89.4)	75(72.8)	196(98)	<0.00001
	Lose weight	177(58.4)	49(47.6)	128(64)	0.008
	Stop smoking	163(53.8)	31(30.1)	132(66)	<0.0001
	Start or do more exercise	191(63)	67(65)	124(62)	0.602
	Advice to use DASH diet	162(53.4)	43(41.7)	119(59.5)	0.005
Number of Antihypertensives	1 medication	59(19.5)	21(20.4)	38(19)	0.891
	2 medications	207(68.3)	73(70.9)	134(67)	0.578
	≥3 medications	37(12.2)	9(8.7)	28(14)	0.254
Antihypertensive medication	Enalapril	191(63)	77(74.8)	114(57)	0.156
	Hydrochlorothiazide	120(39.6)	47(45.6)	73(36.5)	0.922
	Metoprolol	7(2.3)	3(2.9)	4(2)	0.922
	Amlodipine	132(43.6)	56(54.4)	76(38)	0.009
	Nifedipine	92(30.4)	37(35.9)	55(27.5)	0.168
	Furosemide	39(12.9)	12(11.7)	27(13.5)	0.783
	Losartan	3(0.9)	1(0.9)	2(1)	0.881

HCP: health care providers; **hyperthyroidism, intermittent claudication, angina, and stroke; ¥: hospitalized and/or visit clinics during the follow up for acute cases; ***other than HIV/AIDS; DASH: a dietary approach for stopping hypertension.

### 3.4 Blood pressure control of participants

Among hypertensive PLHIV, the consecutive twelve months follow-up average was 145.73 ± 9.40 mmHg for systolic and 98.59 ± 11.33 mmHg for diastolic BP. However, it was 136.87 ± 12.65 mmHg systolic and 89.46 ± 13.45 mmHg diastolic BP for hypertensive HIV negative participants. The consecutive follow-up average BP of the participants revealed that the overall prevalence of uncontrolled BP was 62(60.2%) for PLHIV and 106(53%) among HIV negative patients (p = 0.283).On each quarter, except the fourth, there was a statistically significant difference in BP control between HIV (+) and HIV (−) either on diastolic or systolic blood (Table 5).

**Table 5:** Quarterly and overall blood pressure control status among study participants.

Blood pressure level	Participants group	Follow uptime				
		1st quarter	2nd quarter	3rd quarter	4th quarter	Overall
SBP (mmHg) [Mean ± SD]	HIV (+)	150.22 ± 8.31	146.74 ± 9.46	144.04 ± 7.43	141.91 ± 7.05	145.73 ± 9.40
	HIV (−)	142.12 ± 9.07	136.90 ± 11.13	138.11 ± 8.27	134.34 ± 9.15	136.87 ± 12.65
DBP(mmHg) [Mean ± SD]	HIV (+)	99.32 ± 11.67	95.52 ± 9.46	94.31 ± 10.34	91.22 ± 6.03	98.59 ± 11.33
	HIV (−)	94.24 ± 10.89	92.12 ± 11.64	92.07 ± 8.84	88.22 ± 9.11	89.46 ± 13.45
SBP>140 mmHg & DBP>90 mmHg^£^; n (%)	HIV (+)	78(75.7)	72(69.9)	68(66)	66(64.1)	62(60.2)
	HIV (−)	112(56)	110(55)	110(55)	109(54.5)	106(53)
	P-value	0.001	0.017	0.084	0.139	0.283
SBP>140 mmHg^£^; n (%)	HIV (+)	76(73.8)	70(67.9)	68(66)	62(60)	–
	HIV (−)	111(55.5)	110(55)	106(53)	102(51)	–
	P-value	0.002	0.040	0.040	0.161	–
DBP>90 mmHg^£^; n (%)	HIV (+)	72(69.9)	64(62.1)	66(64.1)	54(52.4)	–
	HIV (−)	106(53)	104(52)	102(51)	98(49)	–
	P-value	0.006	0.092	0.040	0.571	–

£: number of participants; SBP: Systolic blood pressure; DBP: diastolic blood pressure; HIV (+): HIV positive; HIV (−): HIV negative.

### 3.5 Self-management behavior of participants

The mean ± SD total score of hypertensive HIV negative [HIV (−)] patients' perception of self-management behaviors was 2.27 ± 1.39 (ranging from 1.35 to 3.85). For HIV positive [HIV (+)] hypertensive patients the overall perception of self-management behaviors was 2.10 ± 0.77 (ranging from 1.20 to 3.20) (p = 0.122). The mean ± SD score of five categories of self-management for HIV (+) hypertensive participants were as follows: self-integration (1.98 ± 1.87), self-regulation (2.10 ± 1.27), self-integration with the health professional and significant others (1.99 ± 1.45), self-monitoring (2.15 ± 0.69), and adherence to the recommended regimen (1.96 ± 0.65).For hypertensive HIV negative patients the mean ± SD score for each category of self-management behaviors was; self-integration (2.14 ± 1.26), self-regulation (2.17 ± 0.75), self-integration with the health professional and significant others (2.43 ± 0.74), self-monitoring (2.92 ± 0.82), and adherence to the recommended regimen (2.85 ± 0.69). Based on the categorized levels of self-management, the results showed that the total self-management behaviors and each dimension of self-management behaviors were at a moderate level. However, on the dimension of self-regulation, interaction with HCP and significant others and adherence to recommended regimen participants living with HIV showed a low level of self-management of the score (Table 6).

**Table 6:** The mean ± SD score of hypertension self-management behavior of study participants.

Self-management behavior score		HIV (+)	HIV (−)	P-value
Total	Min	1.20	1.35	0.122
	Max	3.20	3.85	
	Mean ± SD	2.10 ± 0.77	2.27 ± 1.39	
	Level	Moderate	Moderate	
Self-integration	Min	1.14	1.24	0.045
	Max	3.24	4.00	
	Mean ± SD	1.98 ± 1.87	2.14 ± 1.26	
	Level	Low	Moderate	
Self-regulation	Min	1.00	1.22	0.089
	Max	3.68	4.00	
	Mean ± SD	2.10 ± 1.27	2.17 ± 0.75	
	Level	Moderate	Moderate	
Interaction with HCP and significant others	Min	1.10	1.36	0.006
	Max	3.46	3.62	
	Mean ± SD	1.99 ± 1.45	2.43 ± 0.74	
	Level	Low	Moderate	
Self-monitoring	Min	1.11	1.33	0.141
	Max	3.80	4.00	
	Mean ± SD	2.15 ± 0.69	2.92 ± 0.82	
	Level	Moderate	Moderate	
Adherence to the recommended regimen	Min	1.00	1.40	0.051
	Max	3.46	4.00	
	Mean ± SD	1.96 ± 0.65	2.85 ± 0.69	
	Level	Low	Moderate	

HCP: health care professionals; Min: Minimum; Max: Maximum; SD: standard deviation.

### 3.6 Factors associated with blood pressure control

On bivariate analysis, the following correlates were significantly associated with uncontrolled blood pressure: an increased waist circumference [CHR: 4.93; 95% CI:(2.21–11.61); p < 0.001], presence of chronic disease co-morbidity [CHR:4.27; 95% CI: (2.04–8.95);p = 0.003], alcohol use history [CHR:1.27;95% CI: (1.06–1.51);p = 0.01], HIV co-infection[CHR:8.81; 95% CI: (2.41–1.20); p = 0.001], early age of hypertension diagnosis[COR: 3.05;95% CI: (1.27–7.31);p = 0.012], not frequently used fruits & vegetables [CHR:2.60;95% CI: (1.06–6.37);p = 0.038], not frequently perform physical exercise [CHR: 2.46;95% CI: (1.13–5.80);p = 0.024], frequently used foods in high fats [CHR:2.29;95% CI: (1.01–5.20);p = 0.047]. In the multivariate Cox-regression analysis, an increased waist circumference [AHR: 2.16; 95% CI: (1.58–5.18);p = 0.021], chronic disease co-morbidity [AHR: 3.94; 95%CI: (2.24–8.74);p = 0.046], alcohol use history [AHR:1.26; 95%CI:(1.08–2.23);p = 0.031], HIV co-infection[AHR:3.06;95%CI: (1.93–11.34);p = 0.042], infrequent use of fruits & vegetables [AHR:3.77;95%CI: (1.34–10.57);p = 0.012], infrequent engagement on physical exercise [AHR:3.48;95%CI: (1.48–8.17);p = 0.004], frequent use of foods in high fats [AHR:2.56;95%CI: (1.25–5.25);p = 0.011] were the significant and independent predictors of uncontrolled blood pressure (Table 7).

**Table 7:** Socio-demographic, behavioral and clinical factors associated with uncontrolled blood pressure.

Variables		Blood pressure status		CHR(95% CI)	P-Value	AHR(95%CI)	P-value
		Controlled	Uncontrolled				
Gender	Male	67(22.2)	91(30)	0.85(0.56–1.30)	0.450	–	
	Female	68(22.4)	77(25.4)	1			
Age (years)	18–35	30(9.9)	16(5.3)	1		1	
	36–50	53(17.5)	72(23.7)	1.12 (0.93–1.35)	0.240	1.206 [0.579–2.512]	0.580
	51–65	38(12.5)	57(18.8)	1.29 (0.67–2.50)	0.440	1.637 [0.763–3.513]	0.626
	≥66	14(4.6)	23(7.6)	2.14(0.5–71.60)	0.220	1.209 [0.426–3.433]	0.862
Residence	Rural	83(27.4)	93(30.7)	1.04 (1.08–0.86)	0.052	2.17 (1.02–4.96)	0.080
	Urban	5217.2)	75(24.7)	1		1	
Marital status	Single	16(5.3)	21(6.9)	1		1	
	Married	75(24.7)	101(33.4)	2.09 (0.47–4.38)	0.062	1.93 (0.60–2.86)	0.341
	Divorced	24(7.9)	27(8.9)	1.06 (0.24–2.42)	0.080	1.83 (0.16–3.21)	0.122
	Widowed	20(6.6)	19(6.3)	1.19 (0.68–0.98)	0.142	2.77 (0.96–4.99)	0.134
Educational status	Cannot read and write	36(11.9)	51(16.8)	0.40 (0.22–0.80)	0.073	2.90 (1.02–5.19)	0.033
	Primary school	46(15.2)	87(28.7)	0.47 (0.03–1.90)	0.311	1.64 (1.61–4.39)	0.053
	Secondary school	25(8.3)	23(7.6)	0.71 (0.12–2.56)	0.102	1.60 (0.89–2.42)	0.141
	College and above	18(5.9)	7(2.3)	1		1	
Job/Occupation	Government employee	13(4.3)	14(4.6)	1		1	
	Non- gov't employee	7(2.3)	6(1.9)	0.96(0.35–2.72)	0.950	1.24 (0.04–0.74)	0.961
	Self employed	82(27.1)	97(32)	0.75(0.27–1.85)	0.490	1.05 (0.05–0.76)	0.576
	Unemployed	33(10.8)	51(16.8)	1.12 (0.91–1.39)	0.280	1.43 (0.12–0.84)	0.151
Living situation	Living with immediate family	98(32.4)	136(44.8)	1		1	
	Living with extended family	29(9.6)	23(7.6)	0.35(0.14–1.02)	0.115	1.03 (0.20–0.98)	0.412
	Living alone	5(1.6)	9(3)	0.56(0.25–1.23)	0.142	0.28 (0.06–0.86)	0.321
Waist Circumference(cm)	Normal	110(36.3)	91(30)	1		1	
	Above the normal	25(8.3)	77(25.4)	4.93(2.21–11.61)	<0.001	2.16 (1.58–5.18)	0.021
Chronic diseases comorbidity	Yes	29(9.6)	60(19.8)	4.27 (2.04–8.95)	0.003	3.94 (2.24–8.74)	0.046
	No	106(35)	108(35.6)	1		1	
Smoking history	Yes	55(18.2)	61(20.1)	1.09(0.90–1.31)	0.370	–	
	No	80(26.4)	107(35.3)	1			
Alcohol use history	Yes	26(8.6)	43(14.2)	1.27 (1.06–1.51)	0.010	1.26 (1.08–2.23)	0.031
	No	109(36)	125(41.2)	1		1	
Chat chewing	Yes	37(12.2)	30(9.9)	0.91 (0.72–1.15)	0.430	–	
	No	98(32.3)	138(45.6)	1			
HIV/AIDS status	HIV (+)	41(13.5)	62(20.5)	8.81(2.41–1.20)	0.001	3.06 (1.93–11.34)	0.042
	HIV (−)	94(31)	106(35)	1		1	
Family history of HTN	Yes	42(13.8)	47(15.5)	1.21(0.97–1.52)	0.190	1.03 (0.20–0.98)	0.211
	No	93(30.7)	121(40)	1		1	
Early age of HTN diagnosis^¥^	Yes	38(12.5)	83(27.4)	3.05 (1.27–7.31)	0.012	3.64 (0.01–13.16)	0.069
	No	97(32)	85(28.1)	1		1	
Frequently used fruits & vegetables *	Yes	117(38.6)	80(26.4)	1		1	
	No	18(6)	88(29)	2.60 (1.06–6.37)	0.038	3.77 (1.34–10.57)	0.012
Frequently perform physical exercise*	Yes	109(36)	41(13.5)	1		1	
	No	26(8.6)	127(41.9)	2.46 (1.13–5.80)	0.024	3.48 (1.48–8.17)	0.004
Frequently used foods in high fats*	Yes	35(11.5)	43(14.2)	2.29 (1.01–5.20)	0.047	2.56 (1.25–5.25)	0.011
	No	100(33)	125(41.3)	1		1	
Number of antihypertensives	1 medication	37	22	1.51(0.77–2.95)	0.268		
	≥2 medications	113	131	1			

¥: Age hypertension diagnosed ≤40 years; *3 times per week; HTN: Hypertension; HIV/AIDS: Human immune deficiency virus; AIDS: Acquired immune deficiency syndrome;HIV (+): HIV positive; HIV (−): HIV negative.

## 4 Discussions

To the best knowledge of the authors, this is the first paper addressing theclinical care, level of blood pressure control and correlates among hypertensive PLHIV and human immunodeficiency virus (HIV) negative patients in Ethiopia. Working toward the optimization of health systems for chronic care delivery is very crucial in the management of high blood pressure. We found a huge gap in the delivery of clinical care of hypertension in our study population, specifically among hypertensive PLHIV.

Studies in the USA showed that PLHIV received poorer care for their coexisting conditions than did those without HIV. Besides, little is known about the clinical care of patients with HIV and co-morbidity [28]. The data related to chronic disease(s) comorbidity on anindividual's progression along the continuum of care is much scarcerin low and middle-income countries [29,30]. Our study aimed to fill this gap by studying the care continuum among hypertensive PLHIV and HIV negative patients. Furthermore, this study assessed the effect of HIV on hypertension care among patients.

We found a high rate of uncontrolled hypertension (60.2%)among hypertensive PLHIV and 53% among hypertensive HIV negative patients. Several factors have been implicated for the poor control of hypertension in Africa, which are generally related to deficiencies in the healthcare system, non-adherence to medication regimens by patients and physicians' inertia to optimize treatment of hypertension [31,32]. Several studies have documented the unavailability of antihypertensive drugs as well as non-adherence to clinic visits by patients as a result of lack of transportation and time [33]. Poor treatment outcomes in Africa has been documented extensively [34,35]. In comparison to our current result, higher levels of control of hypertension were reported in the USA [36] and Canada [37]. This is not a surprise as the management of hypertension is costly due to its chronic nature. High-income countries have been recording successes in the reduction of the burden related to hypertension and other non-communicable diseases [38].

Our study revealed that HIV infection had a significant association with, and is a predictor of, uncontrolled blood pressure. This may be related to pill burden which leads to non-adherence, inflammatory condition from HIV infection and cytokines effect on blood vessels, fragmentation of clinical care of HIV and hypertension [39]. From the previous integration initiatives, we know that integrated TB and HIV care led to decreased HIV and TB-associated morbidity and mortality [40]. Co-location of services is associated with fewer delays in starting ART and greater uptake of ART among HIV/TB co-infected patients [41,42] versus referral to a separate facility for TB or HIV care. Likewise, patients with dual diagnoses of HIV and hypertension were less likely to achieve normal blood pressure over time than those patients receiving care for hypertension only. This could be explained by the disintegrated care for the two services. The patients return to the chronic care clinic for the refill and encounter different health care professionals at each clinic. Therefore, as a lesson learned from Malawi [43], Uganda [44], Cambodia [45] hypertension care should be integrated into the HIV clinic to prevent redundant visits and as well as comprehensive evaluation and management of each patients for the double burden of chronic diseases.

In our study, we found that the hazards of uncontrolled BP was almost four times likely among hypertensivescomorbid with chronic disease [AHR: 3.94; 95%CI: (2.24–8.74); p = 0.046]. Hypertension frequently coexists with obesity, diabetes, hyperlipidemia, or the metabolic syndrome; the identification and management of these risk factors is an important part of the overall management of hypertensive patients. This is in line with multiple studies across the globe.Research outputs from UK [46], France [47], Czech Republic [48], USA [[49], [50], [51]], Korea [52], sub-Saharan Africa [53]showed the unfavorable effect of multimorbidity on the treatment outcome of hypertension. This might be related to their effect on the adherence(prescribed regimen and health education), higher health expenditure, and also patients with these comorbidities are more likely to require combination therapy, yet physicians are often reluctant to adjust the number and doses of medications to achieve target BP.

Despite the presence of several useful pharmacologic and non-pharmacologic methods to hypertension management, hypertension control is still poor, with less controlled blood pressure witnessed. Hypertension self-management behaviors comprising medication adherence, self-blood pressure monitoring, and lifestyle modifications involving diet, exercise, and tobacco are critical components of recommended hypertension treatment and have been associated with significant improvements in hypertension control [54]. The current study showed that infrequent engagement on physical exercise, alcohol use, infrequent use of fruits& vegetables, increased waist circumference, frequent use of foods in high fats were statistically associated with uncontrolled hypertension.

The effects of physical activity on blood pressure are well documented through different studies [55,56]. In their meta-analysis of 93 randomized controlled trials, Cornelissen and Smart found an association between different types of training and a reduction in systolic and diastolic pressure, with pre and hypertensive individuals experiencing bigger reductions than those participants with normal blood pressure [57]. In the Australian Longitudinal Study on Women, authors observed that the risk for hypertension decreased with increases in the total number of hours of metabolic time spent doing exercise, with little difference between moderate and vigorous activity [58]. Similarly, a dose/response association was found among usual cyclists in the UK, showing greater reductions beyond the physical activity’ recommendations [59]. The roles of a dietary approach to stop hypertension (DASH) diets on blood pressure control are investigated well and have robust shreds of evidence [[60], [61], [62], [63], [64]]. The DASH diet encourages the patients to reduce the sodium in diet and foods in high fats and eat a variety of foods rich in nutrients that help lower blood pressure, such as potassium, calcium, and magnesium.

In our current study, we highlighted the self-management practice of hypertensive HIV (+) and HIV (−) participants. On the self-management domains of HSMBQ, there was a significant difference in self-integration (p = 0.045), interaction with HCP and significant others (p = 0.006) and adherence to the recommended regimen (p = 0.051) between the two groups. It is obvious that chronic diseases are prolonged, have a fluctuating course, and are rarely cured completely [65]. HIV meets several chronic disease criteria: uncertain course, affect adherence to the prescribed regimen, some degree of stigma, changes in roles and relationships, and psychological distress which will affect the chronic co-morbid diseases outcomes [66]. This all might affect the self-care management of HIV infected patient integration to their treatment follow up, interaction with health care professionals and others. As well it will significantly affect the adherence to antihypertensive(s). The WHO includes self-management as a best practice to improve clinical care and outcomes for chronic conditions [67]. Programs that educate and support patients to manage their conditions have demonstrated success in achieving improved health outcomes [68]. Self-management programs are effective in reducing morbidity, lessening requirements for acute medical services, and improving lung function and quality of life [69]. Similarly, hospital visits are reduced when self-management training is provided to people [70]. Thus, self-management programs not only improve patient outcomes but also reduce the burden on healthcare system resources and capacities.

### 4.1 Strength and limitations of the study

The major strength of this study was its prospective study design and the continuous follow up of each participant every month for the outcome which will decrease bias and missed data. The prospective data collection allowed us to collect accurate relevant data from our study participants. The weakness of the study was a small sample size and one hospital report which affect its external validity.

## 5 Conclusion

The rate of uncontrolled blood pressure is significantly higher in the HIV infected population. There was a gap in the clinical care of hypertension in terms of hypertension self-management among hypertensive HIV positive patients. Our study showed that overall self-management behavior with hypertension was at a moderate level. An increased waist circumference, chronic disease co-morbidity, alcohol use history, HIV co-infection, infrequent use of fruits & vegetables, infrequent engagement on physical exercise, frequent use of foods in high fats were the significant and independent determinants of uncontrolled blood pressure. It also highlights the need for better integration of hypertension care within the HIV clinical setting.

## Availability of data and materials

The data sets generated during and/or analyzed during the current study are available from the corresponding authors on reasonable request.

## Ethical approval

Ethical clearance & approval was obtained from the institution review board (IRB) of Jimma University with the reference number of IHRPGC/2098/10.It was based on the 1964 Helsinki declaration and its later amendments or comparable ethical standards.

## Sources of funding

The only funder for the study was 10.13039/501100005068Jimma University. The funding body did not have any role in study design, data collection, and data analysis, interpretation of data or in writing the manuscript.

## Authors’ contributions

TM contributes to the design of the study, analysis, interpretation and writes up of the manuscript. TM, LC, and ZM made the data analysis and interpretation of the data. LC and ZM contributed to the design of the study, drafting, and edition of the manuscript. All authors critically revised the manuscript and have approved the final manuscript.

## Trial registry number

⁃ Name of the registry: Research registry, https://www. researchregistry.com
⁃ Unique Identifying number or registration ID: **researchregistry5258**
⁃ Hyperlink to the registration (must be publicly accessible):https://www.researchregistry.com/register-now#user researchregistry/registerresearchdetails/5de12d34b674f70015dfb25b/

## Consent

Not applicable. No person's details, images or videos were used in this study.

## Provenance and peer review

Not commissioned, externally peer reviwed.

## Declaration of competing interest

The authors declared that they have no competing interests.

## Abbreviations

AHR: Adjusted Hazard Ratio
ART: Antiretroviral Therapy
BP: Blood Pressure
CCCC: Comprehensive Chronic Care Clinic
CD4: Cluster Of Differentiation 4
CHR: Crude Hazard Ratio
CI: Confidence Interval
CVD: Cardiovascular Disease
DBP: Diastolic Blood Pressure
HIV: Human Immunodeficiency Virus
HR: Hazard Ratio
HTN: Hypertension
JUMC: Jimma University Medical Center
NCD: Non-Communicable Disease
NNRTIs: Non-Nucleoside Reverse Transcriptase Inhibitors
SBP: Systolic Blood Pressure
SD: Standard Deviation
STEPs: Stepwise Approach to Surveillance
WC: Waist Circumference
WHO: World Health Organization
